# Long-term epidemiological patterns of pediatric infectious diseases in an urban district of Shanghai, 2015–2023

**DOI:** 10.3389/fped.2026.1777444

**Published:** 2026-05-29

**Authors:** Hong Tong, Huizhen Wang, Chenxi Wang, Xingyu Chen, Biao Meng, Shujun Zeng, Chenyan Jiang, Ziyu Qian

**Affiliations:** 1 Emergency Management Office, Shanghai Xuhui Center for Disease Prevention and Control, Shanghai Xuhui Health Inspection Agency, Shanghai, China; 2 Department of Acute Infections Disease Prevention, Shanghai Xuhui Center for Disease Prevention and Control, Shanghai Xuhui Health Inspection Agency, Shanghai, China; 3 Division of Infectious Disease Control and Prevention, Shanghai Municipal Center for Disease Control and Prevention, Shanghai, China

**Keywords:** children, epidemiological trends, infectious diseases, joinpoint regression, post-pandemic period

## Abstract

**Background:**

Understanding temporal and epidemiological patterns of pediatric infectious diseases is essential for developing targeted prevention strategies. This study investigated long-term incidence trends and epidemiological characteristics of notifiable infectious diseases among children in Xuhui District, Shanghai, from 2015 to 2023.

**Methods:**

Surveillance data for children aged 0–17 years were obtained from the National Notifiable Disease Reporting System (NNDRS). Joinpoint regression was applied to identify temporal trends and significant inflection points. Age-specific distributions were analyzed and seasonal patterns were visualized using radar charts.

**Results:**

From 2015 to 2023, a total of 27,940 pediatric cases involving 23 notifiable infectious diseases were reported, corresponding to an average annual incidence of 2,421.68 per 100,000 children. Joinpoint regression identified a significant inflection point in 2021. Overall incidence declined during 2015–2021 (APC = −11.71%, 95% CI: −30.10 to −1.50, *P* = 0.029) and increased sharply thereafter (APC = 141.05%, 95% CI: 34.40–244.50, *P* < 0.001). When COVID-19 cases were excluded, no significant long-term trend was observed (APC = −4.2%, 95% CI: −23.60 –20.80, *P* = 0.71), indicating that the apparent post-2021 increase was driven primarily by COVID-19 notifications rather than a generalized resurgence of other pediatric infections. Disease-specific trajectories varied: influenza showed a pronounced post-pandemic surge, despite the absence of a significant long-term monotonic trend, whereas varicella, mumps, and scarlet fever continued to decline; in contrast, other infectious diarrhea showed a sustained upward trend (APC = 8.00%, 95% CI: 2.60–13.70, *P* = 0.003). Over time, the age distribution shifted toward school-aged children, with a significantly increasing proportion of cases occurring among those aged ≥4 years (*χ*^2^_trend_ = 1475.594, *P* < 0.01).

**Conclusion:**

Pediatric infectious disease epidemiology in Xuhui District underwent substantial changes across the pre-pandemic and post-pandemic periods. The sharp rise after 2021 was largely attributable to COVID-19, rather than a uniform rebound of all infectious diseases. Distinct temporal patterns across respiratory, enteric, and other infections underscore the importance of pathogen-specific transmission characteristics and age-related exposure in shaping long-term trends. These findings highlight the need for targeted, age-appropriate prevention strategies and sustained surveillance in the post-pandemic era.

## 1 Introduction

Pediatric infectious diseases remain a leading cause of morbidity and mortality worldwide. Owing to developing immune systems and frequent exposure in high-contact settings such as schools and childcare facilities, children play a central role in the transmission of infectious diseases, underscoring the importance of continuous pediatric surveillance (1). The COVID-19 pandemic and the widespread implementation of non-pharmaceutical interventions (NPIs), including school closures, mask-wearing, and mobility restrictions, substantially suppressed the circulation of multiple childhood pathogens (2, 3). During this prolonged period of reduced exposure, the proposed concept of “immunity debt” suggests that population-level susceptibility may increase as routine immune stimulation is interrupted (4, 5). Consistent with this notion, post-pandemic rebounds involving multiple pathogens have been reported in several countries following the relaxation of COVID-19 control measures (6–9).

Despite these global observations, longitudinal analyses spanning the full continuum from the pre-pandemic period through strict suppression and into the post-pandemic phase remain limited. Such evidence is particularly scarce in densely populated East Asian urban settings, where concentrated school-based interactions and high population mobility may substantially amplify transmission dynamics (10). Xuhui District in Shanghai offers a valuable context for addressing this gap. As a central metropolitan district characterized by high residential density and substantial cross-district student mobility (11), Xuhui experienced prolonged and stringent public health measures during the COVID-19 pandemic, extending through late 2022. This setting provides a unique opportunity to examine how extended suppression of pathogen circulation influences subsequent epidemiological patterns among children.

In this study, we analyzed surveillance data on notifiable infectious diseases among children aged 0–17 years in Xuhui District from 2015 to 2023. We aimed to quantify long-term incidence trends and identify temporal inflection points using Joinpoint regression, while characterizing age-specific distributions and seasonal patterns to contextualize epidemiological changes observed in the post-pandemic era.

## 2 Materials and methods

### 2.1 Data sources

Data on notifiable infectious diseases were obtained from the National Notifiable Disease Reporting System (NNDRS), an internet-based real-time surveillance platform administered by the Chinese Center for Disease Control and Prevention. The system covers all levels of medical and public health institutions across the country and provides timely and standardized surveillance data.

We extracted records of all notifiable infectious disease cases reported between 1 January 2015 and 31 December 2023 among children aged 0–17 years whose registered residential address was located in Xuhui District, Shanghai. All data were fully de-identified prior to analysis and included standardized variables such as sex, age, occupation (e.g., student, preschool child), date of onset, date of diagnosis, disease classification, and residential subdistrict. Case diagnoses followed the national diagnostic criteria for notifiable infectious diseases issued by the National Health Commission of China. Population data for Xuhui District from 2015 to 2023 were obtained from the Xuhui District Statistical Yearbook. No personally identifiable information was included in the dataset. This study was based on anonymized surveillance data and publicly available population statistics and was exempt from ethical review according to institutional policies.

### 2.2 Statistical analysis

Temporal trends in pediatric infectious disease incidence were assessed using NNDRS data from 2015 to 2023. Annual incidence rates were calculated as the number of cases per 100,000 children aged 0–17 years, using the corresponding annual population estimates for children in Xuhui District as the denominator. Publicly available district statistics showed only modest variation in the population aged 0–17 years between 2020 and 2022, suggesting limited impact of denominator instability on the annual incidence estimates. Joinpoint regression analysis was used to detect potential turning points in annual incidence trends. Given that only nine annual observations were available, the maximum number of joinpoints was restricted to one to reduce the risk of overfitting and maintain model interpretability. The optimal model was selected using the Bayesian information criterion (BIC), and a log-linear model was applied to estimate the annual percent change (APC) for each identified trend segment. Seasonal patterns were visualized using radar charts based on monthly incidence data for each calendar year. Differences in monthly case distributions across the pre-pandemic (2015–2019), pandemic control (2020–2022), and post-pandemic (2023) periods were compared using chi-square tests for each major disease.

Data management and statistical analyses were performed using Microsoft Excel 2016, R software (version 4.2.1), and the Joinpoint Regression Program (version 4.8.0.1; National Cancer Institute). A two-sided significance level of *α* = 0.05 was applied, and *P* < 0.05 was considered statistically significant.

## 3 Results

### 3.1 Overall epidemiological profile

From 2015 to 2023, a total of 27,940 notifiable infectious disease cases were reported among children aged 0–17 years in Xuhui District, Shanghai, corresponding to an average annual incidence rate of 2,421.68 per 100,000 children (range: 717.27–6,556.82 per 100,000).

Based on the cumulative ranking of the seven most frequently reported diseases over the study period (Table 1), influenza ranked first overall, followed by hand, foot and mouth disease (HFMD), varicella, COVID-19, scarlet fever, other infectious diarrhea, and mumps.

**Table 1:** Ranking of major notifiable infectious diseases among children in Xuhui District, Shanghai, 2015–2023.

Diseases	Influenza	HFMD	Varicella	COVID-19	Scarlet fever	Other infectious diarrhea	Mumps
Total 2015–2023	1	2	3	4	5	6	7
2015	6	1	2	/	3	4	5
2016	6	1	2	/	3	4	5
2017	5	2	1	/	3	4	6
2018	6	1	2	/	3	4	5
2019	1	2	3	/	4	5	6
2020	1	4	2	7	5	3	6
2021	4	1	2	7	6	3	5
2022	3	5	2	1	7	4	6
2023	1	2	4	3	6	5	7

“/” indicates that the disease was not reported or not included in the notifiable infectious disease surveillance system for the corresponding year. COVID-19 cases were first reported in 2020 and were therefore not ranked in earlier years.

Temporal changes in disease ranking revealed distinct patterns across pathogens. Influenza rose to the leading position from 2019 onward, whereas it ranked lower prior to that year. HFMD consistently ranked second in most years, indicating a persistently high burden among children in Xuhui District. Varicella ranked among the top two or three diseases between 2015 and 2019 but declined in rank after 2020, falling to fourth place by 2023. COVID-19, first reported in 2020, showed a rapid increase in ranking, reaching first place in 2022 before declining to third in 2023. In contrast, scarlet fever exhibited a gradual downward shift in ranking, decreasing from third in 2015 to sixth in 2023. The rankings of other infectious diarrhea and mumps remained relatively stable throughout the study period, generally fluctuating between fourth and seventh place.

### 3.2 Joinpoint regression analysis

Across the study period, the incidence of notifiable infectious diseases demonstrated marked temporal heterogeneity. Joinpoint regression identified a statistically significant inflection point in 2021 (*P* < 0.05). Between 2015 and 2021, the overall incidence showed a fluctuating but declining trend (APC = −11.71%, 95% CI: −30.10 to −1.50, *P* = 0.029). However, from 2021 to 2023, a sharp resurgence was observed, with an annual percent change of 141.05% (95% CI: 34.40–244.50, *P* < 0.001).

To account for the substantial increase in COVID-19 cases reported after policy adjustments in late 2022, overall incidence excluding COVID-19 was also examined. When COVID-19 cases were removed, the total incidence showed no significant long-term trend (APC = −4.2%, 95% CI: −23.60–20.80, *P* = 0.71), indicating that the pronounced post-2021 increase was largely attributable to the surge in COVID-19 notifications rather than a uniform rise across other pediatric infectious diseases.

Joinpoint regression analysis was conducted for several major pediatric infectious diseases, excluding COVID-19 due to its limited temporal span (data available only from 2020 onward). The results indicated heterogeneous temporal trends across disease categories.

**Figure 1:**
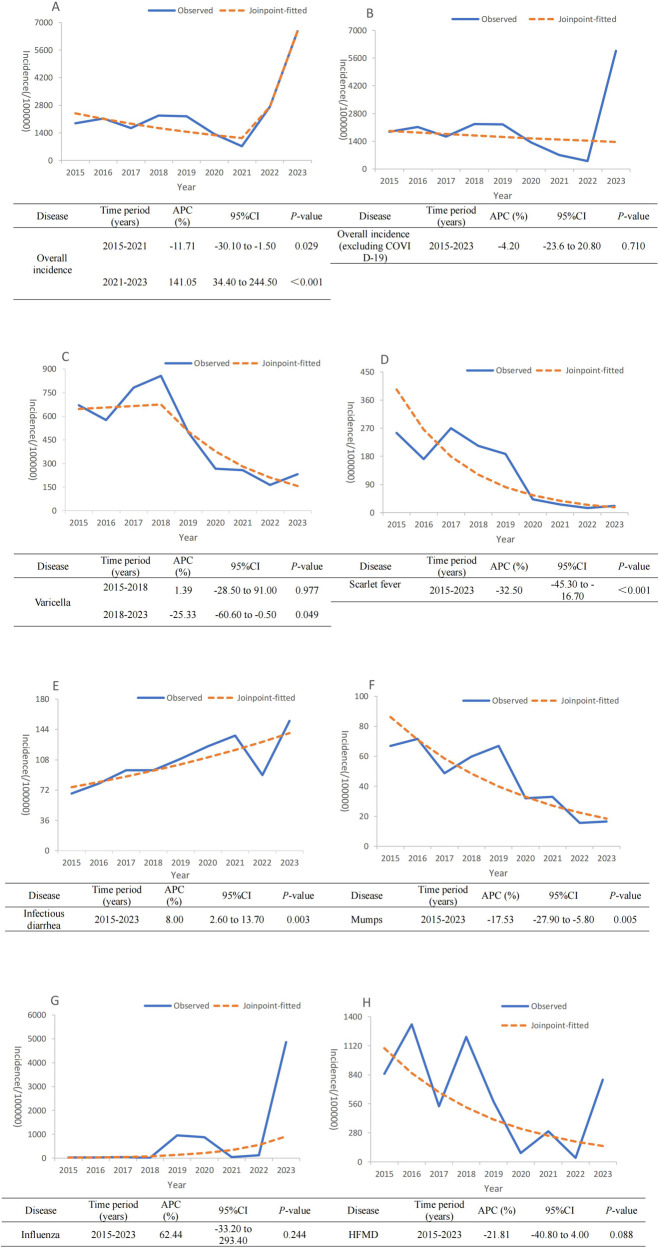
Annual trends in the incidence of infectious diseases among children aged 0–17 in Xuhui District, Shanghai, 2015–2023 (**(A)** overall incidence **(B)** overall incidence (excluding COVID-19) **(C)** varicella **(D)** scarlet fever **(E)** other infectious diarrhea **(F)** mumps **(G)** influenza **(H)** HFMD).

1. Varicella
  A significant inflection point was identified in 2018. From 2015 to 2018, the incidence remained stable (APC = 1.39%, 95% CI: −28.50–91.00, *P* = 0.977). From 2018 to 2023, however, a significant decreasing trend was observed (APC = −25.33%, 95% CI: −60.60 to −0.50, *P* = 0.049).
2. Scarlet fever
  Scarlet fever exhibited a sustained and statistically significant decreasing trend throughout 2015–2023 (APC = −32.50%, 95% CI: −45.30 to −16.70, *P* < 0.001), with no inflection point detected.
3. Mumps
  Mumps also demonstrated a persistent downward trend during the study period (APC = −17.53%, 95% CI: −27.90 to −5.80, *P* = 0.005).
4. Other infectious diarrhea
  Unlike the above diseases, other infectious diarrhea showed a significant and continuous increasing trend (APC = 8.00%, 95% CI: 2.60–13.70, *P* = 0.003).
5. HFMD and influenza
  Both HFMD and influenza displayed pronounced interannual fluctuations with clear seasonal periodicity. Due to this substantial variability, neither disease exhibited a statistically significant long-term trend (HFMD APC = −21.81%, 95% CI: −40.80–4.00, *P* = 0.088; influenza APC = 62.44%, 95% CI: −33.20–293.40, *P* = 0.244). However, influenza incidence in 2023 was substantially higher than the mean annual pre-pandemic level during 2015–2019 (4863.43 vs. 216.23 per 100,000), representing an approximately 22.49-fold increase, indicating a pronounced post-pandemic resurgence. Detailed Joinpoint estimates are presented in Figure 1.

### 3.3 Epidemiological characteristics of incidence trends

#### 3.3.1 Age distribution of cases

**Figure 2:**
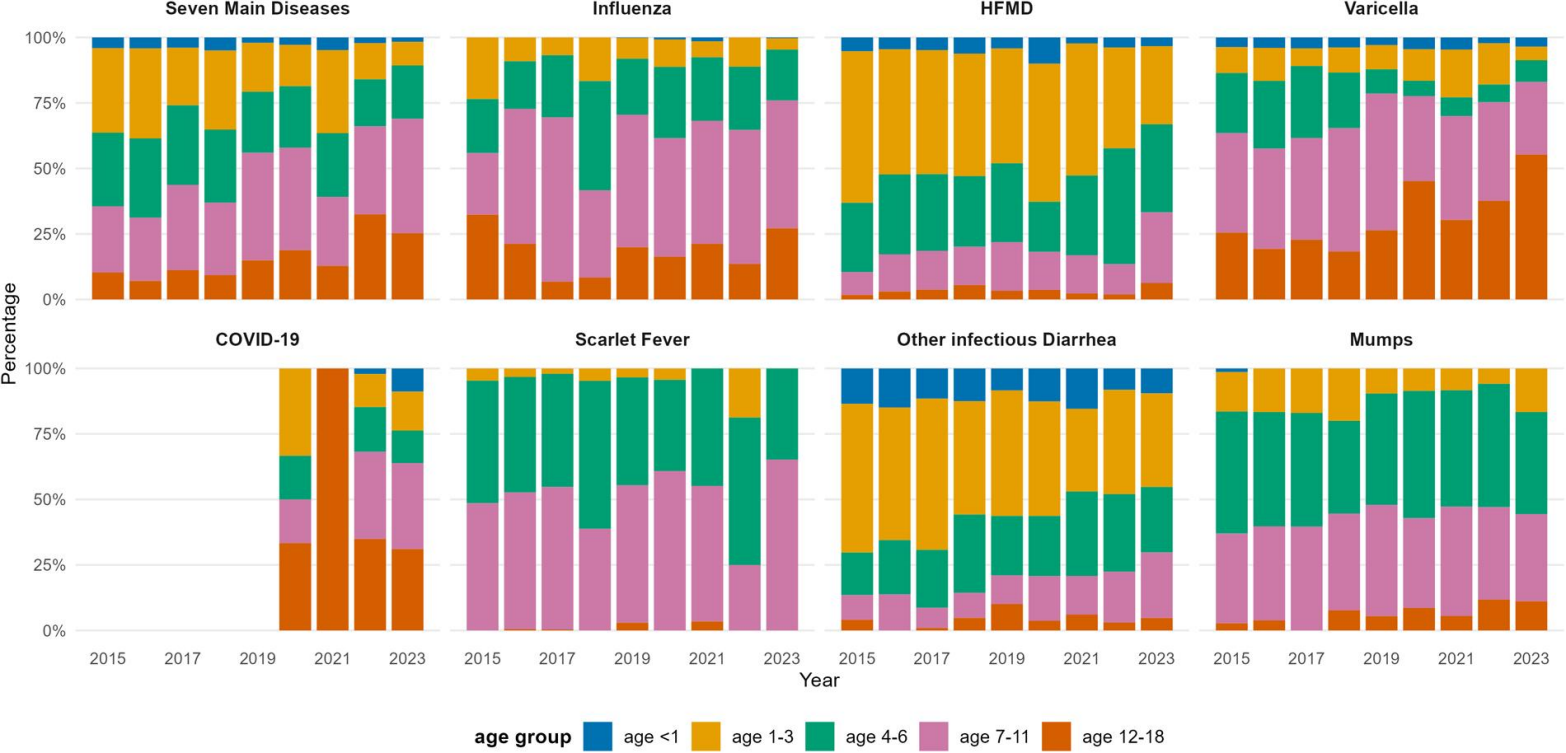
Age distribution of major infectious diseases among children in Xuhui District, Shanghai, from 2015 to 2023.

Children were classified into five groups on the basis of children's growth and development as well as the structure of school system in Shanghai: infants (less than or equal to 1 year old), toddlers (2–3 years), preschoolers (4–6 years), primary school students (7–11 years), and secondary students (12–17 years) (12, 13). Age-specific distributions varied markedly across infectious diseases (Figure 2). Overall, respiratory infections tended to affect older children, whereas enteric infections were concentrated in younger age groups. Influenza and scarlet fever cases were predominantly observed among school-aged children, with the highest incidence in those aged 7–11 years. Similarly, mumps primarily affected children aged 4–6 and 7–11 years. In contrast, HFMD and other infectious diarrhea mainly occurred among younger children, particularly those aged 1–3 years. Varicella showed a broader age distribution, although cases were most frequent in children aged 4–6 and 7–11 years. COVID-19 cases reported during 2022–2023 were largely concentrated among school-aged children, especially those aged 7–11 years and 12–17 years. Across the seven major infectious diseases, the proportion of cases occurring in children aged ≥ 4 years increased significantly over time (*χ*^2^_trend_ = 1475.594, *P* < 0.01).

#### 3.3.2 Seasonal distribution

**Figure 3:**
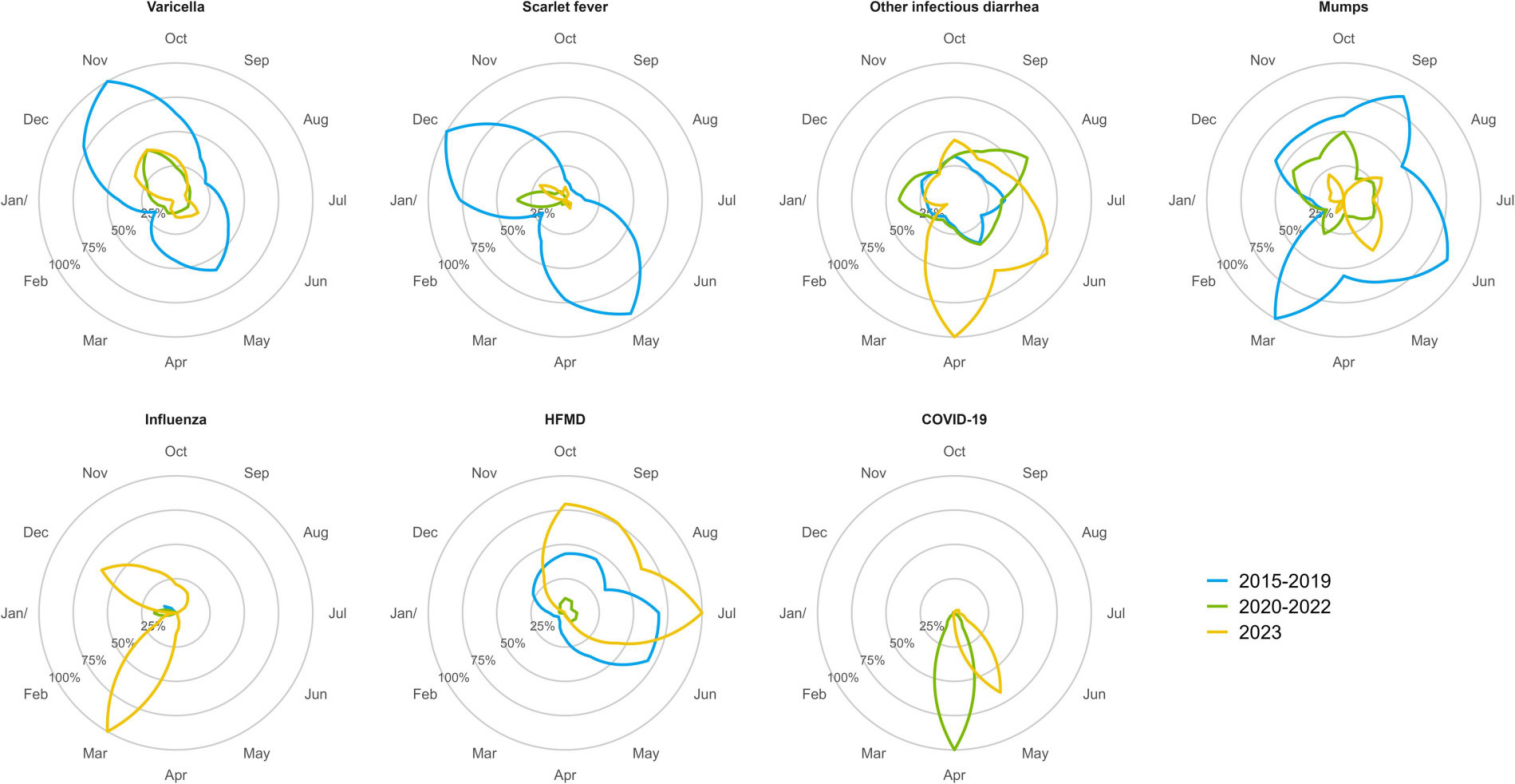
Radar charts illustrating the monthly distribution of major pediatric infectious diseases in Xuhui District, Shanghai, across three periods: the pre-pandemic period (2015–2019), the pandemic control period (2020–2022), and the post-pandemic year (2023).

Seasonal patterns of major pediatric infectious diseases in Xuhui District varied across the pre-pandemic (2015–2019), pandemic control (2020–2022), and post-pandemic (2023) periods (Figure 3). Before the pandemic, most respiratory and contact-transmitted diseases exhibited clear seasonal structures, whereas enteric infections showed stable warm-season predominance.

During 2015–2019, influenza demonstrated a pronounced winter–spring peak, while varicella and scarlet fever displayed bimodal distributions with elevated activity in spring and early winter. Mumps showed a narrower seasonal pattern, peaking mainly in spring. In contrast, HFMD and other infectious diarrhea consistently occurred during warmer months, with HFMD peaking in early summer.

During the pandemic control period (2020–2022), the seasonal amplitudes of most respiratory and contact-transmitted diseases were markedly attenuated, resulting in flattened monthly distributions. In comparison, enteric infections retained clear seasonality, with HFMD and other infectious diarrhea continuing to peak between May and October.

In 2023, seasonal patterns of respiratory infections re-emerged prominently. Influenza showed a sharp winter peak that exceeded pre-pandemic seasonal levels, while varicella, scarlet fever, and mumps exhibited partial restoration of their pre-pandemic seasonal profiles. The seasonal distribution of HFMD and other infectious diarrhea remained largely unchanged, with persistent summer–autumn predominance. COVID-19 cases reported during 2022–2023 were highly concentrated in winter months, with a pronounced peak in late 2022.

These visual patterns were supported by chi-square tests, which showed significant differences in monthly case distributions across the three periods for influenza (*P* < 0.001), mumps (*P* = 0.035), varicella (*P* < 0.001), scarlet fever (*P* < 0.001), HFMD (*P* < 0.001), and other infectious diarrhea (*P* < 0.001).

## 4 Discussion

This study provides a comprehensive assessment of pediatric infectious diseases in Xuhui District, Shanghai, from 2015 to 2023, highlighting pronounced temporal and age-specific heterogeneity in the context of the COVID-19 pandemic. Overall incidence followed a clear V-shaped trajectory, characterized by a sustained decline from 2015 to 2021 and a sharp increase thereafter. The initial decline coincided with the period of stringent NPIs, including universal masking, school closures, and mobility restrictions, which substantially curtailed transmission opportunities for multiple pathogens (14–16). The subsequent increase observed in 2022–2023 likely reflects a combination of resumed population mixing, changes in healthcare-seeking behavior, and a marked rise in COVID-19 notifications following national policy adjustments. Importantly, when COVID-19 cases were excluded, no significant long-term trend was detected, indicating that the apparent post-2021 surge was driven primarily by SARS-CoV-2 rather than a generalized resurgence of other pediatric infectious diseases. This finding provides important context for interpreting the pathogen-specific patterns described below.

A salient finding of this study is the divergent temporal trajectories across different pathogen groups. Among respiratory infections, influenza exhibited a pronounced increase in incidence in the post-pandemic period (17, 18), despite the absence of a statistically significant long-term monotonic trend in Joinpoint regression. This apparent discrepancy reflects the intrinsic epidemiological characteristics of influenza, which is defined by strong seasonality and substantial interannual variability rather than steady linear change. Consequently, sharp increases concentrated within a short time window, such as the marked surge observed in 2023, may not be captured as statistically significant long-term trends (19, 20). Nevertheless, the magnitude of the post-pandemic influenza peak clearly exceeded pre-pandemic seasonal levels, indicating a meaningful epidemiological rebound following prolonged suppression of viral circulation (21, 22). In contrast, several other pediatric infectious diseases—including varicella, mumps, and scarlet fever—demonstrated sustained declines throughout the study period. These patterns are consistent with the continued effects of routine school-based infection control measures and, in the case of varicella, the sustained decline after 2018 may be partly associated with the inclusion of varicella vaccine in Shanghai's immunization programme (23), which likely improved vaccine accessibility and enhanced population-level protection. Previous evidence from Shanghai reported that the two-dose coverage among target children reached 99.6% in 2018, providing indirect support for the role of immunization in shaping varicella trends (24).

Enteric infections displayed a distinct and comparatively stable epidemiological pattern. Unlike the episodic rebounds observed for respiratory infections, the continuous increase in other infectious diarrhea began prior to the COVID-19 pandemic and persisted thereafter (25). This temporal consistency indicates that factors beyond changes in social mixing were involved. Potential contributors include increased healthcare-seeking awareness for gastrointestinal symptoms, the high etiological diversity of enteric pathogens with limited cross-protective immunity (26), and enhanced surveillance sensitivity resulting from strengthened reporting systems in schools and childcare institutions (27). Together, these findings suggest that the long-term upward trend in enteric infections reflects pathogen-specific transmission characteristics and surveillance dynamics rather than transient post-pandemic effects.

Age-specific analyses further demonstrated a shifting disease burden toward older children, with an increasing proportion of cases occurring among those aged ≥ 4 years. Younger children were predominantly affected by hand, foot and mouth disease and infectious diarrhea, whereas influenza, scarlet fever, and COVID-19 were more common among school-aged populations (28, 29). These patterns likely reflect age-dependent differences in immune maturation, behavioral exposure, and social contact structures, emphasizing the need for age-tailored prevention strategies that distinguish between childcare and school-based settings. Beyond long-term incidence trends, seasonal analyses revealed stable and disease-specific temporal patterns. Respiratory infections, including influenza and COVID-19, consistently peaked during the winter–spring months, whereas enteric infections—such as hand, foot and mouth disease and other infectious diarrhea—predominantly occurred in the warmer summer–autumn period (30). These seasonal distributions were largely preserved across the pre-pandemic, suppression, and post-pandemic phases, although the amplitude of seasonal peaks varied substantially between years. Such stability underscores the enduring role of climatic and behavioral factors in shaping pediatric infectious disease transmission and provides predictable windows for targeted public health preparedness.

Several limitations should be acknowledged. First, the NNDRS is a passive surveillance system, and its completeness depends on healthcare-seeking behavior, clinical recognition, diagnostic access, and timely reporting. During the COVID-19 pandemic, changes in healthcare utilization, reduced access to routine medical services, delayed diagnosis, and possible reporting delays may have reduced case ascertainment. Second, changes in diagnostic practice during and after the COVID-19 period may also have influenced pathogen-specific detection patterns. In particular, the expanded use of multiplex PCR assays, broader respiratory pathogen screening, and heightened clinical vigilance may have increased the detection of certain non-SARS-CoV-2 pathogens, such as influenza. Third, interpretation of “other infectious diarrhea” should be made in the context of its broad and heterogeneous surveillance definition, which includes multiple enteric infections caused by viruses, bacteria, and protozoa. Because these pathogens differ in transmission patterns, seasonality, and sensitivity to behavioral and public health interventions, the observed temporal changes in this category may reflect the combined effects of multiple agents, altered exposure patterns, and variation in diagnostic and reporting practices over time. In addition, the absence of pathogen-specific genotyping or serotyping data limited further exploration of strain-level dynamics underlying the observed trends.

## 5 Conclusion

This nine-year analysis demonstrates substantial shifts in the epidemiology of pediatric infectious diseases in Xuhui District across the pre-pandemic, NPIs, and post-pandemic periods. The observed V-shaped trajectory underscores the profound impact of COVID-19 mitigation measures on pathogen circulation, while the sharp increase after 2021 was driven predominantly by a surge in COVID-19 notifications rather than a generalized resurgence of other pediatric infections. Marked heterogeneity was observed across disease categories. Influenza exhibited a pronounced post-pandemic rebound following the restoration of population mixing, whereas varicella, mumps, and scarlet fever continued to decline, reflecting the sustained effects of routine immunization and school-based infection control. In contrast, other infectious diarrhea showed a persistent long-term increase independent of the pandemic, suggesting the influence of pathogen-specific characteristics and evolving surveillance sensitivity. Age-specific shifts toward school-aged children further highlight the role of social contact patterns and institutional settings in shaping transmission dynamics. Together, these findings emphasize that post-pandemic pediatric infectious disease control requires pathogen- and age-specific strategies, including strengthened respiratory infection prevention in schools, enhanced hygiene measures in childcare settings, and continued high vaccination coverage, supported by robust and adaptive surveillance systems.

## Data Availability

The data analyzed in this study is subject to the following licenses/restrictions: The data analyzed in this study were obtained from the National Notifiable Disease Reporting System and are not publicly available due to data protection regulations, but may be accessed from the corresponding author upon reasonable request and with permission from the relevant authorities. Requests to access these datasets should be directed to wanghz191@outlook.com.
